# The Landscape of Tumor-Infiltrating Immune Cells in Feline Mammary Carcinoma: Pathological and Clinical Implications

**DOI:** 10.3390/cells11162578

**Published:** 2022-08-18

**Authors:** Catarina Nascimento, Andreia Gameiro, Jorge Correia, João Ferreira, Fernando Ferreira

**Affiliations:** 1 CIISA—Centro de Investigação Interdisciplinar em Sanidade Animal, Faculdade de Medicina Veterinária, Universidade de Lisboa, Avenida da Universidade Técnica, 1300-477 Lisboa, Portugal; 2 Laboratório Associado Para Ciência Animal e Veterinária (AL4AnimalS), 1300-477 Lisboa, Portugal; 3 Instituto de Medicina Molecular, Faculdade de Medicina, Universidade de Lisboa, Avenida Prof. Egas Moniz, 1649-028 Lisboa, Portugal

**Keywords:** feline mammary carcinoma, tumor microenvironment, TILs, TAMs, cancer model

## Abstract

Feline mammary carcinoma (FMC) shares key molecular and clinicopathological features with human breast cancer. We have herein studied the inflammatory infiltrate of FMC in order to uncover potential therapeutic targets and prognostic markers. To this end, the expression of different markers (CD3, CD4, CD8, CD20, CD56, FoxP3, CD68 and CD163) was analyzed in total, stromal (s) and intratumoral (i) tumor-infiltrating lymphocytes (TILs) and tumor-associated macrophages (TAMs), in 73 feline mammary carcinomas. The results revealed that higher percentages of sCD8^+^ TILs were associated with longer disease-free survival (*p* = 0.05) and overall survival (*p* = 0.021). Additionally, higher percentages of iCD4^+^ TILs correlated with positive lymph node status (*p* = 0.003), whereas CD163^+^ TAMs were associated with undifferentiated tumors (*p* = 0.013). In addition, sCD3^+^ (*p* = 0.033), sCD8^+^ (*p* = 0.044) and sCD68^+^ (*p* = 0.023) immune cells were enriched in triple negative normal-like carcinomas compared to other subtypes. Altogether, our results suggest that specific subsets of immune cells may play a major role in clinical outcome of cats with mammary carcinoma, resembling what has been reported in human breast cancer. These data further support the relevance of the feline model in breast cancer studies.

## 1. Introduction

Breast cancer is the most diagnosed cancer in women, showing a high mortality rate, despite the great progress in breast cancer therapies [1]. Usually, human breast cancer is classified in five molecular subtypes (Luminal A, Luminal B, HER2-positive, Triple negative normal-like and Triple negative basal-like), based on the expression of immunohistochemical markers in cancer cells [2]. Nevertheless, in the last years, several studies have highlighted the importance of the cross talk between cancer cells and immune cell subpopulations present in tumor microenvironment (TME), associated with either pro or anti-tumor activities [3,4,5]. The breast cancer microenvironment is composed by distinct immune cells, including T and B cells, macrophages, dendritic cells and other cell subtypes [6], which can be present at the intratumoral and/or at stromal compartments of TME, being crucial to predict the clinical outcome of breast cancer patients [7,8].

Previous research demonstrated that the presence of tumor-infiltrating lymphocytes (TILs) has an independent positive prognostic value in breast cancer patients [9,10,11]. Indeed, recent studies demonstrated that TILs were more frequently observed in the microenvironment of triple-negative breast cancers (TNBC), with those located in the stromal compartment predicting a longer survival [12,13]. Notwithstanding, the distinct phenotypes of immune cell subpopulations present in TME seem to contribute in different ways to tumor biology. Indeed, the adaptive immune response involves B lymphocytes (CD20^+^) and T cells (CD3^+^), including CD4^+^ T-helper 1 and cytotoxic T lymphocytes (CD8^+^) which counteract tumor development and progression [14,15,16]. Particularly, CD8^+^ TILs have been shown to be a major player as an antitumor component of TME, with stromal CD8^+^ T cell overexpression being associated with a better disease-free survival (DFS) in patients with breast cancer [17]. Conversely, CD4^+^ T-helper 2 and FoxP3^+^ regulatory T cells seem to contribute to tumor immune escape [8,18]. Moreover, NK cells (CD56^+^) are part of the innate immune system and are able to kill cancer cells without prior sensitization [19], predicting a favorable outcome in patients with breast cancer [20]. Accordingly, a group of investigators developed a standardized methodology to evaluate TILs, in order to integrate the analysis of these cells in histopathological practice and clinical trials [12]. In parallel, tumor-associated macrophages (TAMs) are the major component of the innate immunity in TME and can be polarized into two distinct phenotypes: classically activated M1 and alternatively activated M2 macrophages [21]. While M1 macrophages (iNOS-positive) [22] associate with anti-tumor activity, leading to the activation of adaptive immune cells, M2 macrophages (CD163) inhibit immune function, supporting tumor proliferation and metastasis [23]. Additionally, higher levels of TAMs located in the tumor stroma are associated with worse outcome in dogs with mammary carcinoma and in women with breast cancer [24].

Similar to human breast cancer, feline mammary carcinoma (FMC) remains as one of the most common tumors in cat, showing high malignancy and metastasis rate [25,26]. Indeed, FMC has been considered a suitable model of human breast cancer due to the resemblance between the two neoplasms, including histological, clinicopathological and molecular features [27,28,29]. Nonetheless, the TME of FMC is still very poorly studied. Thus, this study aims to: (i) quantify and compare the number of TILs, such as T lymphocytes (CD3^+^), T-helper cells (CD4^+^), cytotoxic T lymphocytes (CD8^+^), B lymphocytes (CD20^+^), NK cells (CD56^+^), FoxP3^+^ regulatory T cells, as well as the number of total TAMs (CD68^+^) and polarized M2 macrophages (CD163^+^), within a collection of 73 FMC; (ii) check for correlations between tumor-infiltrating immune cells and clinicopathological features, in order to identify specific subpopulations that may predict the clinical outcome of cats with mammary carcinoma and (iii) investigate the distribution of the immune populations across the distinct molecular subtypes of FMC.

## 2. Materials and Methods

### 2.1. Animal Population and Tissue Collection

In this retrospective study, a total of 73 cats with spontaneous mammary carcinoma were included, all of whom underwent mastectomy at the Small Animal Hospital of the Faculty of Veterinary Medicine from University of Lisbon. After the surgery, there were two animals that underwent chemotherapy. Tumor samples were collected in accordance with the EU Directive 2010/63/EU. The tissues were embedded in paraffin after fixation in 10% buffered formalin (pH 7.2) during 24–48 h. For each animal enrolled in the study, the following clinicopathological characteristics were recorded: age, reproductive status, contraceptive administration, number, location and size of tumor lesions, histopathological classification, malignancy grade, presence of tumor necrosis, lymphatic invasion, lymphocytic infiltration, cutaneous ulceration, regional lymph node involvement, stage of the disease (TNM system), DFS and OS. The TNM system was assessed using a modification of the World Health Organization’s TNM system used for malignant neoplasms in humans [30].

In this study all the animals were anesthetized before surgical procedures and samples were collected during anesthesia. Therefore, as there was no interference with animal well-being the Commission on Ethics and Animal Wellbeing considered that there was no reason for additional advice. All samples were collected in accordance with the EU Directive 2010/63/EU and national legislation (DL113/2013). In addition, informed consent was obtained from all the cat owners. All methods were carried out in accordance with relevant guidelines and regulations.

### 2.2. Immunohistochemistry Validation

The immunohistochemistry (IHC) assessment of immune cell subpopulations was validated using human tonsil and feline lymph node samples as controls. After tissue section (Microtome Leica RM2135, Newcastle, UK), deparaffinization, rehydration and antigen retrieval were performed using a PT-Link module (Dako, Agilent, Santa Clara, CA, USA), by boiling glass slides in citrate buffer pH 6.0 (for CD20, CD56, FoxP3 and CD68 labelling) or Tris-EDTA buffer pH 9.0 (for CD3, CD4, CD8 and CD163 labelling) from Dako, at 96 °C. Then, slides were cooled for 30 min at room temperature (RT) and rinsed twice for 5 min in distilled water. Thereafter, sections were blocked with Peroxidase Block Novocastra Solution (Novacastra, Leica Biosystems, Newcastle, UK) during 15 min at RT, followed by two washing steps with PBS pH 7.4, and Protein Block Novocastra Solution (Leica Biosystems) during 10 min. After two washes with PBS for 5 min, tissue slides were incubated with the following antibodies: CD3 (clone A0452, dilution 1:100, Dako), CD4 (clone 10C12, dilution 1:100, Abcam, Cambridge, UK), CD8 (ab4055, dilution 1:200, Abcam), CD20 (clone CD20 (P), dilution 1:100, Biocare Medical, Pacheco, CA, USA), CD56 (clone 1G4, dilution 1:100, Abcam), FoxP3 (236A/E7, dilution 1:100, Abcam), CD68 (clone KP1^+^ C68/684, dilution 1:1000, Abcam) and CD163 (clone GHI/61, 20 μg/mL, Abcam). After incubation, each tissue section was washed with PBS 2× for 5 min and subsequently treated with the Post-Primary Reagent (Leica Biosystems) for 30 min at RT and with the Novolink Polymer (Leica Biosystems) for 30 min. Afterwards, sections were stained with DAB Chromogen Solution (Leica Biosystems) for 5 min and nuclei were counterstained with Gills hematoxylin (Merck, Branchburg, NJ, USA). Slides were dehydrated in an ethanol gradient and mounted with Entellan mounting medium (VWR International, Radnor, PA, USA).

### 2.3. Immunofluorescence Staining, Image Collection and Evaluation

Once each antigenic detection was optimized for IHC analysis, immunofluorescence (IF) assays were performed in FMC tissues. Staining was carried out by implementing the same procedures as those in IHC, until the incubation step with the primary antibodies. Then, after two washes with PBS, tissue sections were incubated 30 min at RT with the corresponding secondary antibodies: donkey anti-mouse IgG H&L Alexa Fluor 488 (ab150105, 1:1000, Abcam) or donkey anti-rabbit IgG H&L Alexa Fluor 568 (ab175470, 1:1000, Abcam). From this step forward, samples were protected from light and washed with PBS 2× for 5 min. Finally, tissue sections were counterstained with flourished mounting medium containing DAPI (Abcam) for 5 min. Slides were cover slipped and observed in a Leica DFC340 FX fluorescence microscope (Leica Microsystems).

Tissue sections were blindly evaluated from three individual fields randomly selected at high resolution (200× magnification, total area = 1.15 mm^2^) to determine the percentage of CD3^+^, CD4^+^, CD8^+^, CD20^+^, CD56^+^, FoxP3^+^, CD68^+^ and CD163^+^-cells in the immune cell populations. The percentage of the different cellular population was obtained by dividing the number of stained cells by the number of total immune cells. The absolute number of cells were counted using the open-source Java-based image processing program software Image J (version 2.1.0, National Institutes of Health, Bethesda, MD, USA). For each tumor, the average of stained immune cells in the three evaluated fields was calculated. Necrotic areas and technical artifacts were avoided. Positively stained cells in contact with tumor cells or within the tumor cell nests were defined as intratumoral, while positively stained cells in the interstitial stroma surrounding cancer cells were defined as stromal.

### 2.4. Statistical Analysis

The statistical software program IBM SPSS (version 25; Armonk, NY, USA) was used to perform the computations for all analyses. The GraphPad Prism version 8.1.2 (GraphPad Software, San Diego, CA, USA) was used to plot the graphs. The cut-off values were defined by the 25th percentile. Univariate analysis was carried out using the chi-square test or Fisher’s exact test to determine the significance of differences between percentage of immune cell subpopulations and clinicopathological features. The DFS and OS were determined by the Kaplan-Meier method and compared by the log-rank test. Univariate analyses were performed using Cox proportional hazard model. The Kruskal-Wallis test and Dunn’s multiple comparisons post-test were applied to compare the percentage of positive cells between the distinct FMC molecular subtypes. Results were presented as median values. Two-tailed *p*-values of less than 0.05 were considered statistically significant.

## 3. Results

### 3.1. Clinical and Histopathological Characteristics

In the entire cohort, the average age at diagnosis was 11.7 ± 0.3 years (range 7–18 years), the median age was 11.3 ± 2.8 years, and the interquartile range was 4. The DFS was 9.6 ± 1.1 months (95% CI: 7.4–11.7 months) and the overall survival (OS) was 14.5 ± 1.3 months (95% CI: 11.8–17.2 months). The clinicopathological characteristics are summarized in Table 1. None of the animals was excluded from the study.

### 3.2. CD3^+^ T Cells Are the Predominant Infiltrating Cell Type in Feline Mammary Carcinoma

**Figure 1:**
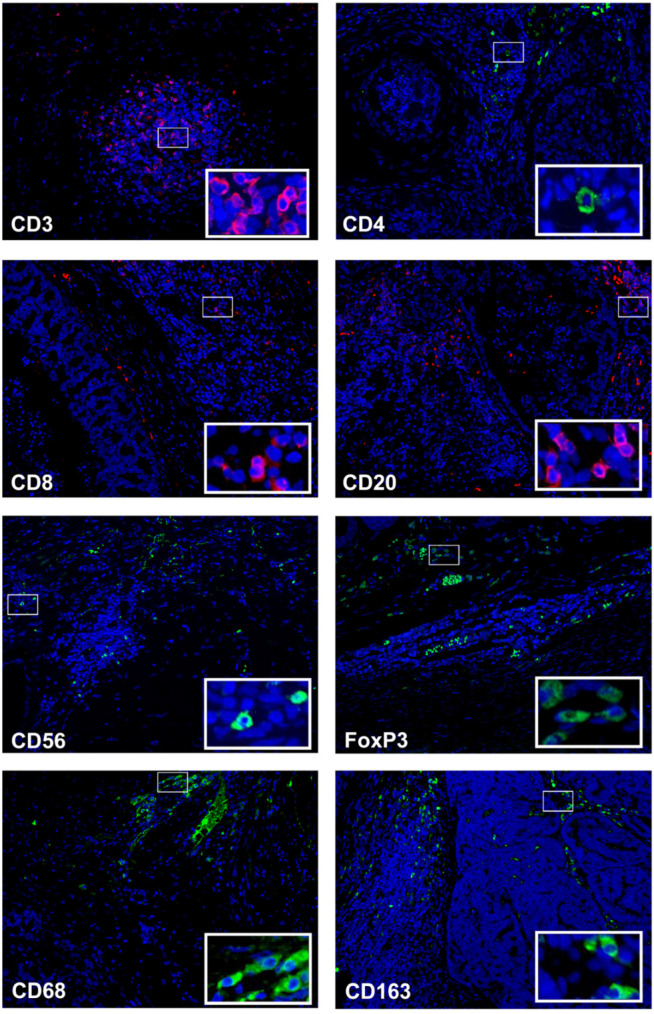
CD3^+^ tumor infiltrating lymphocytes are the most common immune cells in feline mammary tumor samples. Representative micrographs of immunofluorescence of mammary tumors stained for CD3^+^, CD4^+^, CD8^+^, CD20^+^, CD56^+^, FoxP3^+^, CD68^+^ and CD163^+^; insets depict high-power magnification of positive cells. Original magnification, 200×.

Immunofluorescence analyses revealed a heterogeneous distribution of the inflammatory cells infiltrate in FMC (*n* = 73). In the entire cohort, CD3^+^ T lymphocytes were the most common subset of immune cells, followed by B lymphocytes (CD20^+^), with average percentages of 17.6% and 14.4%, respectively. Infiltration by CD4^+^, CD8^+^, CD56^+^ and FoxP3^+^ TILs was identified within tumors in lower percentages, with CD8^+^ T lymphocytes being the most abundant of the four T cell-subsets. In parallel, approximately 32% of the tumor-associated macrophages (CD68^+^) showed an M2-polarized subtype (CD163^+^). Regarding the localization of the immune cell subpopulations, they were mainly found in the stromal compartment, with the exception of CD8^+^ T lymphocytes and CD68^+^ macrophages that were primarily found in the intratumoral compartment. The mean and median values of total, stromal and intratumoral, as well as representative images of each immune cell subtypes are depicted in Table 2 and Figure 1.

In addition, several statistically associations were found between the different subsets of immune cells and clinicopathologic parameters. Indeed, higher percentages of total CD3^+^, sCD3^+^, total CD4^+^, sCD4^+^ TILs, and total CD163^+^ TAMs were significantly associated with malignancy grade III tumors (*p* = 0.016, *p* = 0.031, *p* = 0.035, *p* = 0.043 and *p* = 0.013, respectively), with higher percentages of sCD163^+^ macrophages showing a *p* value of *p* = 0.051. In parallel, higher percentages of total CD3^+^ TILs and of sCD8^+^ T cells were negatively correlated with metastasis (*p* = 0.021 and *p* = 0.017, respectively), contrasting with tumors showing higher percentages of iCD3^+^ and sCD56^+^ TILs (*p* = 0.019 and *p* = 0.049, respectively). In addition, higher percentages of iCD4^+^ T cells were correlated with positive lymph node status (*p* = 0.003), and total CD8^+^ TILs were significantly associated with tumor necrosis (*p* = 0.011). All statistical correlations between each immune cell subpopulation and clinicopathological parameters are summarized in the Supplementary Table S1.

### 3.3. Stromal Densities of CD8^+^ Tumor-Infiltrating Lymphocytes Are Prognostic Markers for Feline Mammary Carcinoma

**Figure 2:**
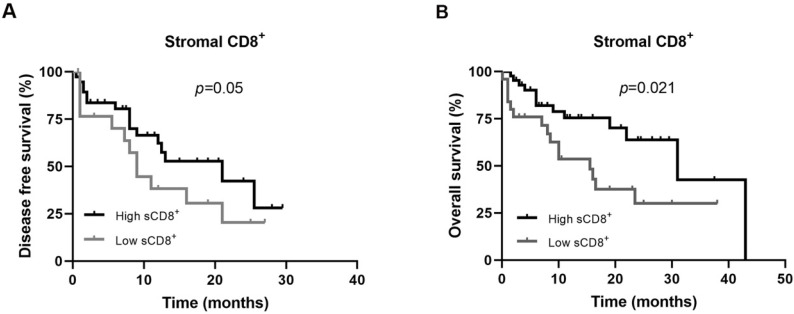
Disease free survival and overall survival of cats with mammary carcinoma according to low or high expression of CD8^+^ receptors in immune cells of tumor microenvironment. Kaplan-Meier curves for association of stromal CD8^+^ T lymphocytes with (**A**) disease free survival, and with (**B**) overall survival.

The Kaplan-Meier analysis showed that the presence of sCD8^+^ TILs in the TME of FMC is a prognostic factor for DFS and OS. Accordingly, cats with mammary carcinoma showing higher percentage of sCD8^+^ TILs in TME had longer DFS and OS, than those with lower percentages of sCD8^+^ TILs (21 ± 6.8 months vs. 8 ± 1.8 months, *p* = 0.05, Figure 2A; 31.0 ± 7.9 months vs. 15.5 ± 4.0 months, *p* = 0.021, Figure 2B).

Additionally, the univariate Cox regression analysis demonstrated that the presence of sCD8^+^ TILs is also a significant predictive prognostic factor for OS in cats with mammary carcinoma (HR: 0.421, CI: 0.197–0.900, *p* = 0.026), and a trend toward significant association for DFS (HR: 0.514, CI: 0.256–1.031, *p* = 0.061). The detailed data of univariate Cox regression analysis are shown in Table 3.

### 3.4. Tumor Infiltration by Stromal CD3^+^ T Cells, CD8^+^ T Lymphocytes and CD68^+^ Macrophages Is Increased in Triple Negative Normal-like Mammary Carcinoma Subtype

**Figure 3:**
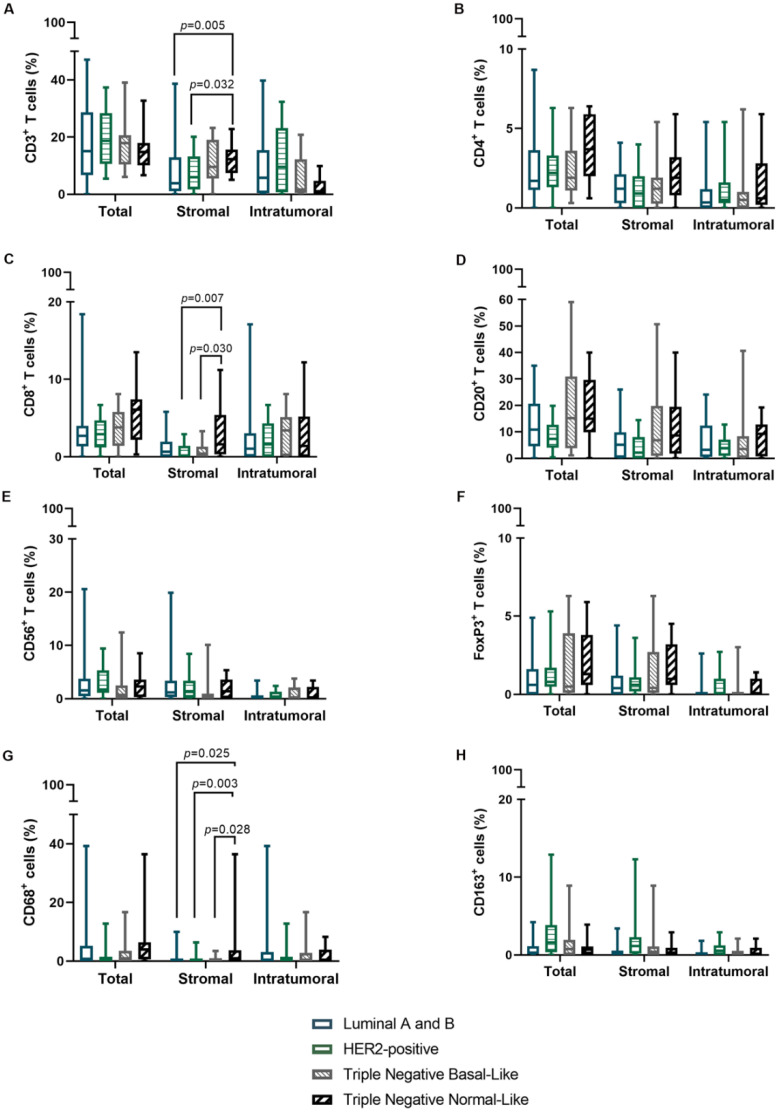
The percentage of stromal CD3^+^ and CD8^+^ TILs and CD68^+^ TAMs is increased in the tumor microenvironment of Triple Negative Normal-Like mammary carcinomas. Box plot with minimum and maximum values of (**A**) CD3^+^, (**B**) CD4^+^, (**C**) CD8^+^, (**D**) CD20^+^, (**E**) CD56^+^, (**F**) FoxP3^+^, (**G**) CD68^+^ and (**H**) CD163^+^ immune cells, according to location and molecular subtype.

Considering the above results, further analysis was performed in order to explore the prevalence of specific TILs and TAMs subpopulations in different molecular tumor subtypes. Results obtained showed that triple negative normal-like tumors featured increased infiltration by sCD3^+^ (*p* = 0.033), sCD8^+^ (*p* = 0.044), and sCD68^+^ (*p* = 0.023) immune cells in comparison to other tumor subtypes (Table 4 and Figure 3).

## 4. Discussion

In the last decade, there has been an ever-growing interest in the investigation of TILs and TAMs present in the breast cancer microenvironment, because of their prognostic and predictive value in the field of immunotherapy, particularly in triple negative breast cancer [31,32,33]. Similar to human breast cancer, FMC remains one of the most frequent causes of cancer-related death in female cats, due to the deficit of effective therapeutic options following mastectomy [34,35]. In this context, and due to the lack of studies in this field, immunofluorescence was used to characterize the phenotype of distinct immune cell subpopulations in the tumor microenvironment of FMC. We found that CD3^+^ T lymphocytes were the predominant population, with the majority being CD8^+^ T cells, suggesting that cytotoxic T cells are fundamental players in TME of FMC; this is in line with previous findings in human breast cancer [32,36,37]. Accordingly, and regarding prognosis, results suggest that a higher proportion of stromal CD8^+^ T cells can be a reliable predictor of favorable outcome in cats with mammary carcinoma. Indeed, a significant association with longer DFS and OS and a negative correlation with tumor metastasis supports sCD8^+^ T cells being key effector cells in anti-tumor immunity, also as reported in human breast cancer [17,36,38,39]. Furthermore, higher percentages of total CD3^+^ T lymphocytes were also negatively associated with tumor metastasis, contrasting with iCD3^+^ T cells, which emphasized the importance of investigating the location of immune cells. In addition, this result is consistent with a previous study in malignant canine mammary tumors, where iCD3^+^ T cells abundance correlated with VEGF expression and angiogenesis [40]. Interestingly, the presence of tumor metastasis was also associated with increased levels of natural killer cells in the stroma compartment of TME. Indeed, in pancreatic cancer it has been reported that cancer cells are able to sequester NK cells in the stroma area, leading to functional deregulation and preventing NK induced cancer cells death [41]. Further studies are needed to address this hypothesis in FMC.

Regarding the tumor malignancy grade, the increased percentage of total CD163^+^ macrophages, especially those located in stroma, were positively associated with poorly differentiated FMC, supporting its negative prognostic role. Indeed, M2-polarized TAMs, have been reported to promote tumor growth in dogs with mammary carcinoma [42,43] and in women with breast cancer [31,44,45,46]. Likewise, the enhanced expression of total and stromal CD3^+^ lymphocytes, and in particular the CD4^+^ T cell-subset, was also correlated with tumor grade III, which raises a possible defense mechanism by stromal T cells against tumor aggressiveness. In contrast, the iCD4^+^ T cells were positively correlated with lymph node involvement, suggesting that its expression may be a poor prognostic factor in FMC, as reported in human breast cancer [47].

Results also demonstrated an association between tumor necrosis and higher percentages of total CD8^+^ T cells. Accordingly, necrotic cells can act as an antigen source enabling the trigger of antigen-specific CD8^+^ T cell responses [48].

Finally, in human breast cancer, the infiltration of immune cells, particularly in the most aggressive tumor subtypes (HER2-positive, triple negative basal-like and normal-like) has been associated with better outcome [49]. Indeed, recent studies in patients with triple negative tumors found that the presence of abundant sCD8^+^ T cells and sCD68^+^ macrophages are associated with good prognosis [50,51]. Accordingly, in this study, the data obtained showed that triple negative normal-like mammary carcinoma had high percentages of sCD3^+^, sCD8^+^ and sCD68^+^ immune cells when comparing with other FMC subtypes. This finding is in accordance with earlier evidence that, despite its aggressive behavior, cats with triple negative normal-like mammary carcinoma had longer OS [25].

In conclusions, this study suggests that stromal and intratumoral lymphocytic and macrophage subset markers might contribute to predict outcomes for cats with mammary carcinoma. Accordingly, our results showed that sCD8^+^ T cells may be a novel, favorable prognostic factor for cats with mammary carcinoma, while iCD3^+^, iCD4^+^, sCD56^+^ T cells and sCD163^+^ macrophages cells associate with worse clinicopathological parameters. Furthermore, high infiltration of sCD3^+^ T cells in combination with sCD8^+^ T lymphocytes and sCD68^+^ TAMs suggested a better outcome in cats with triple negative normal-like tumors. Based on these results, the development of novel strategies to remodel the TME of FMC may prove useful to enhance anti-tumor immune responses. Finally, due to the similarities observed between TME of human and feline mammary carcinoma, the results obtained also emphasize the utility of spontaneous FMC as a model for human breast cancer studies.

## Figures and Tables

**Table 1:** Clinicopathological features of 73 cats with mammary carcinoma enrolled in this study.

Clinicopathological Feature	Number of Animals (%)	Clinicopathological Feature	Number of Animals (%)
Age		Tumor malignancy grade	
<8 years old	2 (2.7%)	I	2 (2.7%)
8–12 years old	38 (52.1%)	II	13 (17.8%)
>12 years old	30 (41.1%)	III	58 (79.5%)
Unknown	3 (4.1%)		
Spayed		Tumor necrosis	
No	42 (57.5%)	No	18 (24.7%)
Yes	28 (38.4%)	Yes	55 (75.3%)
Unknown	3 (4.1%)		
Contraceptive administration		Lymphocytic infiltration	
No	25 (34.2%)	No	15 (20.5%)
Yes	28 (38.4%)	Yes	57 (78.1%)
Unknown	20 (27.4%)	Unknown	1 (1.4%)
Multiple tumors		Tumor ulceration	
No	40 (54.8%)	No	61 (83.6%)
Yes	33 (45.2%)	Yes	12 (16.4%)
Lymph node status		HP classification	
Negative	39 (53.4%)	Tubulopapillary carcinoma	25 (34.2%)
Positive	26 (35%)	Solid carcinoma	11 (15.1%)
Unknown	8 (11%)	Cribriform carcinoma	9 (12.3%)
Tumor size		Papillary-cystic carcinoma	5 (6.8%)
<2 cm	16 (21.9%)	Tubular carcinoma	17 (23.3%)
≥2 cm	57 (78.1%)	Mucinous carcinoma	6 (8.2%)
TNM classification		Molecular subtype	
I	19 (26%)	Luminal A	13 (17.8%)
II	13 (17.8%)	Luminal B	15 (20.5%)
III	34 (46.6%)	HER2-positive	15 (20.5%)
IV	7 (9.6%)	Triple Negative Normal-like	15 (20.5%)
		Triple Negative Basal-like	15 (20.5%)

TNM–Tumor, Node, Metastasis; HP-Histopathological.

**Table 2:** The mean ± standard error of the mean (SEM) and median ± standard deviation (SE) percentages of positive immune cells in the 73 tumors evaluated, according to their tumor location [total, stromal (s) and intratumoral (i)].

Immune Cells	Mean of Positive Cells (%) ± SEM	Median of Positive Cells (%) ± SE
Total CD3^+^	17.6 ± 1.2	16.3 ± 10.3
sCD3^+^	9.3 ± 0.9	7.4 ± 7.8
iCD3^+^	8.3 ± 1.2	4 ± 10.4
Total CD4^+^	2.7 ± 0.2	2.1 ± 1.9
sCD4^+^	1.5 ± 0.2	1.2 ± 1.4
iCD4^+^	1.2 ± 0.2	0.5 ± 1.6
Total CD8^+^	3.9 ± 0.4	2.9 ± 3.5
sCD8^+^	1.4 ± 0.3	0.5 ± 2.1
iCD8^+^	2.6 ± 0.4	1.4 ± 3.2
Total CD20^+^	14.4 ± 1.5	10.9 ± 12.5
sCD20^+^	8.1 ± 1.2	4.8 ± 10.0
iCD20^+^	6.5 ± 0.9	3.7 ± 7.6
Total CD56^+^	2.6 ± 0.4	1.6 ± 3.4
sCD56^+^	1.9 ± 0.4	0.9 ± 3
iCD56^+^	0.7 ± 1.2	0.1 ± 1
Total FoxP3^+^	1.5 ± 0.2	0.8 ± 1.7
sFoxP3^+^	1.2 ± 0.2	0.7 ± 1.4
iFoxP3^+^	0.3 ± 0.1	0 ± 0.7
Total CD68^+^	4.4 ± 0.9	1 ± 8
sCD68^+^	1.4 ± 0.5	0 ± 4.6
iCD68^+^	2.9 ± 0.8	0.3 ± 6.9
Total CD163^+^	1.4 ± 0.3	0.6 ± 2.3
sCD163^+^	1 ± 0.2	0.3 ± 2.1
iCD163^+^	0.4 ± 0.1	0 ± 0.7

**Table 3:** Univariate Cox regression analysis of disease-free survival and overall survival according to immune cell phenotype.

Immune Cell Phenotype	Disease Free Survival		Overall Survival	
	HR (95% CI)	*p*-Value	HR (95% CI)	*p*-Value
Total CD3^+^	0.539 (0.268–1.085)	0.083	1.145 (0.491–2.671)	0.753
sCD3^+^	1.090 (0.511–2.322)	0.824	1.459 (0.611–3.483)	**0.395**
iCD3^+^	2.587 (0.903–7.411)	0.077	1.065 (0.429–2.644)	0.893
Total CD4^+^	1.086 (0.500–2.357)	0.835	1.286 (0.542–3.052)	0.586
sCD4^+^	0.771 (0.363–1.637)	0.499	1.189 (0.502–2.818)	0.693
iCD4^+^	0.959 (0.461–1.995)	0.911	1.262 (0.549–2.902)	0.584
Total CD8^+^	0.864 (0.387–1.929)	0.721	1.031 (0.413–2.575)	0.948
sCD8^+^	0.514 (0.256–1.031)	0.061	0.421 (0.197–0.900)	**0.026**
iCD8^+^	0.771 (0.364–1.632)	0.497	1.199 (0.481–2.991)	0.697
Total CD20^+^	0.954 (0.449–2.027)	0.904	1.425 (0.570–3.558)	0.449
sCD20^+^	1.150 (0.539–2.451)	0.718	2.691 (0.929–7.793)	0.068
iCD20^+^	0.901 (0.396–2.053)	0.804	0.891 (0.355–2.236)	0.806
Total CD56^+^	1.448 (0.595–3.525)	0.415	1.670 (0.577–4.833)	0.344
sCD56^+^	1.838 (0.706–4.783)	0.212	1.861 (0.642–5.393)	0.252
iCD56^+^	0.983 (0.488–1.980)	0.961	1.673 (0.746–3.750)	0.212
Total FoxP3^+^	2.039 (0.714–5.824)	0.183	1.466 (0.505–4.258)	0.482
sFoxP3^+^	2.371 (0.830–6.771)	0.107	1.311 (0.493–3.482)	0.588
iFoxP3^+^	0.915 (0.446–1.876)	0.809	0.901 (0.412–1.970)	0.794
Total CD68^+^	1.571 (0.678–3.641)	0.292	1.091 (0.477–2.496)	0.836
sCD68^+^	0.693 (0.310–1.548)	0.371	0.643 (0.258–1.601)	0.343
iCD68^+^	1.922 (0.860–4.292)	0.111	1.362 (0.605–3.069)	0.456
Total CD163^+^	0.683 (0.314–1.486)	0.337	0.935 (0.375–2.332)	0.886
sCD163^+^	0.704 (0.337–1.472)	0.351	1.322 (0.528–3.310)	0.550
iCD163^+^	0.852 (0.407–1.786)	0.672	1.232 (0.566–2.683)	0.600

HR-Hazard Ratio; CI-Confidence Interval.

**Table 4:** The median ± standard deviation (SE) values of positive immune cell phenotype related proteins according to the feline mammary carcinoma molecular subtype.

Immune Cells	Luminal A and B	HER2-Positive	Triple Negative Basal-like	Triple Negative Normal-like	*p*-Value
Total CD3^+^	15.3 ± 12.9	20.1 ± 9.5	17.9 ± 8.3	14.8 ± 6.5	0.697
sCD3^+^	3.9 ± 8.9	6 ± 6.8	9.6 ± 7.8	12.3 ± 5.5	**0.033**
iCD3^+^	6 ± 12.4	12.5 ± 11.2	1.7 ± 7.7	1.1 ± 3	0.150
Total CD4^+^	1.7 ± 1.9	2.2 ± 1.7	1.8 ± 1.9	3.7 ± 2	0.115
sCD4^+^	1.3 ± 1.3	0.8 ± 1.2	1 ± 1.5	1.9 ± 1.7	0.319
iCD4^+^	0.2 ± 1.5	0.5 ± 1.6	0.6 ± 1.8	0.6 ± 1.8	0.505
Total CD8^+^	2.7 ± 4	2.7 ± 2.1	3.3 ± 2.7	6.1 ± 3.9	0.095
sCD8^+^	0.8 ± 1.5	0.1 ± 1.1	0.4 ± 0.9	1.6 ± 3.6	**0.044**
iCD8^+^	1 ± 3.7	1.5 ± 2.2	2.5 ± 2.7	1.4 ± 3.6	0.763
Total CD20^+^	10.8 ± 11.1	7.3 ± 6.1	12.4 ± 17.6	15 ± 12	0.125
sCD20^+^	5.1 ± 6.7	1.9 ± 4.6	5.9 ± 15.2	8.6 ± 11.1	0.129
iCD20^+^	3.2 ± 7.4	4.2 ± 4.3	2.9 ± 11.5	9.3 ± 6.4	0.908
Total CD56^+^	1.6 ± 4.2	1.7 ± 2.8	0.7 ± 3.4	2.3 ± 2.8	0.366
sCD56^+^	1.2 ± 3.9	1.2 ± 2.6	0.3 ± 2.7	1.4 ± 1.8	0.133
iCD56^+^	0.1 ± 0.9	0.3 ± 0.9	0 ± 1.3	0.2 ± 1.3	0.785
Total FoxP3^+^	0.6 ± 1.3	0.8 ± 1.3	0.5 ± 2	1.3 ± 1.9	0.341
sFoxP3^+^	0.4 ± 1.1	0.6 ± 0.7	0.4 ± 2	1 ± 1.6	0.189
iFoxP3^+^	0 ± 0.6	0 ± 0.8	0 ± 0.1	0 ± 0.5	0.366
Total CD68^+^	0.9 ± 10.5	0.6 ± 3.8	0.9 ± 4.6	4 ± 9	0.344
sCD68^+^	0 ± 2.2	0 ± 1.7	0 ± 1.3	1 ± 9.3	**0.023**
iCD68^+^	0.6 ± 10.3	0.3 ± 3.3	0 ± 4.5	0.3 ± 2.7	0.876
Total CD163^+^	0.3 ± 1.2	1.6 ± 4	0.8 ± 2.3	0.7 ± 1.1	0.149
sCD163^+^	0.2 ± 1	1.2 ± 3.6	0.4 ± 2.3	0.3 ± 0.8	0.195
iCD163^+^	0 ± 0.5	0.5 ± 1	0 ± 0.6	0 ± 0.6	0.485

## Data Availability

The data presented in this study are available on request from the corresponding author. The data are not publicly available as they contain information that could compromise the privacy of future research.

## Supplementary Materials

The following supporting information can be downloaded at: https://www.mdpi.com/article/10.3390/cells11162578/s1, Table S1: (a–d)-Statistical associations between immune cells expression and clinicopathological characteristics of cats with mammary carcinoma (*n* = 73).
