# Supplementary material for: The Landscape of Tumor-Infiltrating Immune Cells in Feline Mammary Carcinoma: Pathological and Clinical Implications
Source: Cells. 2022 Aug 18;11(16):2578. doi: 10.3390/cells11162578 (PMC9406662; doi:10.3390/cells11162578)
Supplement: Supplementary file 1 [file cells-11-02578-s001.zip › cells-1836950-supplementary.pdf]

Table S1. Statistical associations between immune cells expression and clinicopathological characteristics of cats with mammary carcinoma ($n = 73$).

a) Variable	Total CD3			Stromal CD3			Intratumoral CD3			Total CD4			Stromal CD4			Intratumoral CD4		
	Low	High	*p*-value	Low	High	*p*-value	Low	High	*p*-value	Low	High	*p*-value	Low	High	*p*-value	Low	High	*p*-value
Lymph node status																		
Negative	10	29	1.000	9	30	0.953	11	28	0.367	12	27	0.264	11	28	0.367	18	21	**0.003**
Positive	7	19		7	19		4	22		4	22		4	22		2	24	
Stage																		
I	7	12		4	15		6	13		4	15		6	13		6	13	
II	2	11	0.501	4	9	0.854	4	9	0.590	5	8	0.306	5	8	0.297	5	8	0.967
III	7	26		8	25		6	27		6	28		5	29		11	23	
IV	2	5		1	6		1	6		3	4		2	5		2	5	
Tumor size																		
≤2 cm	4	12	1.000	6	10	0.250	2	14	0.394	2	14	0.343	4	12	1.000	4	12	0.647
>2 cm	14	42		11	45		15	41		16	41		14	43		20	37	
Malignancy grade																		
I	2	0		2	0		1	1		2	0		2	0		1	1	
II	5	8	**0.016**	2	11	**0.031**	3	10	0.672	2	11	**0.035**	2	11	**0.043**	7	6	0.166
III	11	46		13	44		13	44		14	44		14	44		16	42	
Tumor necrosis																		
No	4	14	1.000	6	12	0.423	3	15	0.631	5	13	0.969	6	12	0.504	7	11	0.736
Yes	14	40		11	43		14	40		13	42		12	43		17	38	
Tumor ulceration																		
No	17	43	0.273	15	45	0.804	14	46	1.000	15	46	1.000	16	45	0.737	20	41	1.000
Yes	1	11		2	10		3	9		3	9		2	10		4	8	
Metastasis																		
No	4	31	**0.021**	6	29	0.327	13	22	**0.019**	8	28	0.838	6	30	0.197	12	24	1.000
Yes	14	23		11	26		4	33		10	27		12	25		12	25	

Supplementary Table S1 (a-d) - Statistical associations between immune cells expression and clinicopathological characteristics of cats with mammary carcinoma (n=73).

b) Variable	Total CD8			Stromal CD8			Intratumoral CD8			Total CD20			Stromal CD20			Intratumoral CD20		
	Low	High	*p*-value	Low	High	*p*-value	Low	High	*p*-value	Low	High	*p*-value	Low	High	*p*-value	Low	High	*p*-value
Lymph node status																		
Negative	9	30	0.951	13	26	1.000	13	26	0.185	9	29	0.908	12	26	0.646	9	29	1.000
Positive	5	21		9	17		4	22		5	21		6	20		6	20	
Stage																		
I	5	14	0.789	7	12	0.237	5	14	0.346	4	15	0.389	7	12	0.285	4	15	0.262
II	2	11		6	7		5	8		4	8		3	9		2	10	
III	6	28		8	26		6	28		9	25		8	26		11	23	
IV	2	5		4	3		3	4		0	7		0	7		0	7	
Tumor size																		
≤2 cm	3	13	1.000	7	9	0.543	3	13	0.668	2	14	0.394	6	10	0.326	2	14	0.394
>2 cm	12	45		18	39		16	41		15	41		12	44		15	41	
Malignancy grade																		
I	1	1	0.548	1	1	0.864	1	1	0.652	0	1	0.696	0	1	0.135	0	1	0.852
II	3	10		4	9		4	9		4	9		6	7		3	10	
III	11	47		20	38		14	44		13	45		12	46		14	44	
Tumor necrosis																		
No	8	10	**0.011**	6	12	1.000	7	11	0.261	6	12	0.423	6	12	0.530	5	13	0.873
Yes	7	48		19	36		12	43		11	43		12	42		12	42	
Tumor ulceration																		
No	14	47	0.450	22	39	0.685	14	47	0.322	16	44	0.321	17	43	0.273	14	46	1.000
Yes	1	11		3	9		5	7		1	11		1	11		3	9	
Metastasis																		
No	7	29	1.000	7	29	**0.017**	9	27	1.000	7	28	0.671	7	28	0.496	9	26	0.896
Yes	8	29		18	19		10	27		10	27		11	26		8	29	

c) Variable	Total CD56			Stromal CD56			Intratumoral CD56			Total FoxP3			Stromal FoxP3			Intratumoral FoxP3		
	Low	High	*p*-value	Low	High	*p*-value	Low	High	*p*-value	Low	High	*p*-value	Low	High	*p*-value	Low	High	*p*-value
Lymph node status																		
Negative	9	30	0.953	9	30	1.000	20	19	0.167	10	28	0.260	14	28	0.465	28	10	0.094
Positive	7	19		6	20		8	18		3	23		4	22		13	13	
Stage																		
I	4	15	0.871	5	14	0.885	9	10	0.077	3	16	0.151	3	16	0.059	14	5	0.718
II	4	9		4	9		7	6		5	7		6	6		8	4	
III	7	27		7	27		9	25		7	27		8	26		20	14	
IV	2	5		2	5		5	2		0	7		0	7		4	3	
Tumor size																		
≤2 cm	3	13	0.880	4	12	1.000	7	9	1.000	2	14	0.561	2	14	0.394	9	7	0.670
>2 cm	14	43		14	43		23	34		13	43		15	41		37	19	
Malignancy grade																		
I	1	1	0.528	1	1	0.512	2	0	0.116	0	1	0.859	0	1	0.852	1	0	0.667
II	2	11		2	11		7	6		3	10		3	10		9	4	
III	14	44		15	43		21	37		12	46		14	44		36	22	
Tumor necrosis																		
No	3	15	0.657	4	14	1.000	6	12	0.620	4	14	1.000	5	13	0.873	11	7	1.000
Yes	14	41		14	41		24	31		11	43		12	42		35	19	
Tumor ulceration																		
No	14	47	1.000	15	46	1.000	25	36	1.000	13	47	1.000	14	46	1.000	41	19	0.154
Yes	3	9		3	9		5	7		2	10		3	9		5	7	
Metastasis																		
No	10	26	0.536	13	23	**0.049**	15	21	1.000	9	26	0.483	10	25	0.493	24	11	0.576
Yes	7	30		5	32		15	22		6	31		7	30		22	15	

d) Variable	Total CD68			Stromal CD68			Intratumoral CD68			Total CD163			Stromal CD163			Intratumoral CD163		
	Low	High	*p*-value	Low	High	*p*-value	Low	High	*p*-value	Low	High	*p*-value	Low	High	*p*-value	Low	High	*p*-value
Lymph node status																		
Negative	12	27	0.956	26	13	0.874	15	24	0.719	10	29	0.315	13	26	0.383	24	15	0.082
Positive	7	19		16	10		12	14		3	22		5	20		9	16	
Stage																		
I	5	14	0.711	13	6	0.543	6	13	0.615	6	13	0.510	6	13	0.825	12	7	0.703
II	5	8		8	5		6	7		2	11		3	10		6	7	
III	10	24		24	10		16	18		5	27		9	23		16	16	
IV	1	6		3	4		2	5		1	6		1	6		3	4	
Tumor size																		
≤2 cm	2	14	0.189	9	7	0.543	4	12	0.233	1	15	0.237	2	14	0.253	6	10	0.296
>2 cm	19	38		39	18		26	31		13	42		17	38		31	24	
Malignancy grade																		
I	1	1	0.777	2	0	0.383	1	1	0.881	2	0	**0.013**	2	0	0.051	2	0	0.267
II	4	9		7	6		6	7		3	10		4	9		8	5	
III	16	42		39	19		23	35		9	47		13	43		27	29	
Tumor necrosis																		
No	5	13	1.000	12	6	1.000	7	11	1.000	3	15	0.973	4	14	0.845	9	9	1.000
Yes	16	39		36	19		23	32		11	42		15	38		28	25	
Tumor ulceration																		
No	19	42	0.507	43	18	0.112	27	34	0.358	13	46	0.491	16	43	1.000	31	28	1.000
Yes	2	10		5	7		3	9		1	11		3	9		6	6	
Metastasis																		
No	12	24	0.554	20	16	0.118	19	17	0.078	4	31	0.152	7	28	0.317	16	19	0.408
Yes	9	28		28	9		11	26		10	26		12	24		21	15	
